# 4290 Acoustic screening for the “wet voice” in a canine laryngeal model

**DOI:** 10.1017/cts.2020.287

**Published:** 2020-07-29

**Authors:** Anais Rameau

**Affiliations:** 1 Weill Cornell Medicine

## Abstract

OBJECTIVES/GOALS: Early dysphagia detection reduces risk of pulmonary complications, length of hospital stay, and overall healthcare costs. The biggest limitation for early detection has been the lack of a sensitive, reliable, and noninvasive screening tool. The bedside swallow examination may miss silent aspiration in up to 40% of patients. The objective of this study is to evaluate if acoustic parameters can distinguish normal and wet voice in a canine laryngeal model. Ultimately, our goal is to establish whether the sensitivity of the bedside swallow examination can be augmented with the addition of an acoustic screen in humans. METHODS/STUDY POPULATION: Two excised canine larynges were used for laryngeal phonation simulations under six different conditions over 48 hours. Acoustic recordings were obtained while the larynges were in vibration at the phonation threshold pressure. Phonation was recorded under dry conditions and when the laryngeal introitius was covered with low viscosity glycerin (9.5cP) or high viscosity glycerin (950cP), as well as in adducted and abducted conditions. The latter mimics glottic insufficiency seen in presbylarynx or vocal fold paralysis. RESULTS/ANTICIPATED RESULTS: A total of 112 voice samples were generated and analyzed for pitch, sound pressure level (SPL), % shimmer, % jitter, relative average perturbation (RAP), and noise-to-harmonics using PRAAT software. A multivariate regression model showed that pitch, SPL, % shimmer, % jitter and RAP could significantly predict wetness in abducted conditions only. Could you please add numbers and p values? DISCUSSION/SIGNIFICANCE OF IMPACT: This pilot study indicates that classic acoustic perturbation measures distinguish the dry from the wet larynx only in glottic insufficiency condition in an ex vivo canine laryngeal model. Our next step is to study whether non-linear time series analysis and machine learning can differentiate dry and wet phonation in both adducted and abducted conditions in our animal model. CONFLICT OF INTEREST DESCRIPTION: Dr. Anais Rameau is a co-founder and Chief Executive Officer of MyophonX, a wearable device used to restore speech in patients with limited phonation capacity.

